# Pelvic Lymphangioma: The Purpose of a Clinical Case

**DOI:** 10.7759/cureus.103836

**Published:** 2026-02-18

**Authors:** Viktor Baiherych, Tetiana Baiherych, Carlos Nancassa, Petra Pego

**Affiliations:** 1 Internal Medicine, Hospital Distrital Santarém, Santarém, PRT; 2 Medicine, Hospital Distrital Santarém, Santarém, PRT

**Keywords:** benign vascular tumor, pelvic lymphangioma, pelvic mass, urinary retention, vascular malformation

## Abstract

Lymphangioma is a benign vascular malformation characterized by abnormal proliferation of lymphatic vessels, resulting in the formation of lymphatic masses or cysts. Pelvic lymphangiomas may remain asymptomatic for long periods and are often discovered incidentally during imaging studies performed for other reasons. When symptomatic, they may cause manifestations related to compression of adjacent structures, such as abdominal pain and distension, urinary retention, or gastrointestinal symptoms. We report the clinical case of a 37-year-old male patient with no relevant medical history who presented to the emergency department twice within two months due to urinary retention. As part of the etiological study, a computed tomography (CT) scan revealed a large, lobulated pelvic mass of uncertain characterization, raising suspicion for a necrotic mass, fluid collection, lymphangioma, or another cause. Subsequently, pelvic magnetic resonance imaging (MRI) supported the diagnostic hypothesis of lymphangioma. The patient was referred to the Portuguese Institute of Oncology (IPO), Lisbon, where he underwent elective surgery. Histopathological examination confirmed the diagnosis of pelvic lymphangioma.

## Introduction

Lymphangioma is a rare benign vascular malformation resulting from the abnormal proliferation of lymphatic vessels, leading to the formation of cystic or tumoral structures composed of dilated lymphatic channels. Although most frequently diagnosed in the pediatric population, particularly in the cervical and axillary regions, lymphangiomas can also occur in less common locations, such as the abdominal cavity, retroperitoneum, and pelvic cavity [1-3].

The pathophysiology is associated with somatic mutations, most frequently involving the PIK3CA gene, leading to abnormal activation of the PI3K/AKT/mTOR signaling pathway, which drives excessive lymphangiogenesis and aberrant lymphatic vessel development [4].

The clinical presentation is variable: many lymphangiomas remain asymptomatic and are often detected incidentally during imaging studies performed for unrelated reasons or intraoperatively during surgical procedures. Symptom onset is primarily related to the size and location of the lesion, leading to secondary effects from compression of adjacent structures. The most commonly reported symptoms include abdominal pain, nausea, fatigue, and weight loss. When symptomatic, lymphangiomas typically manifest with compressive effects on surrounding organs, resulting in gastrointestinal and/or urinary symptoms [1,3,5].

The differential diagnosis includes benign and malignant tumors, as well as inflammatory or necrotic fluid collections, with CT and MRI being crucial tools for assessment [6]. Definitive diagnosis, however, requires histopathological confirmation [1].

The treatment of choice is complete surgical excision whenever feasible, which is associated with a lower recurrence rate. Nevertheless, the rarity of these lesions, particularly in the pelvic and retroperitoneal locations, continues to pose a significant clinical and diagnostic challenge [3].

## Case presentation

**Figure 1:**
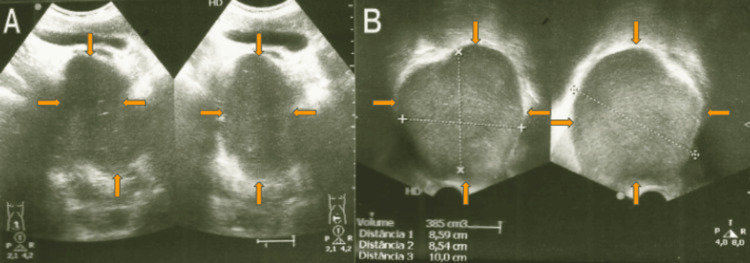
Prostate ultrasound performed via (A) suprapubic and (B) transrectal approaches The prostate demonstrates markedly increased dimensions with bosselated contours. The estimated prostatic weight is approximately 385 g. There is no clear differentiation between the central and peripheral prostatic zones.

We present the case of a 37-year-old male patient with a history of urinary retention for the past two months, requiring presentation to the emergency department (ED) and bladder catheterization. Laboratory studies, including infection parameters, prostate-specific antigen (PSA), and urinalysis, revealed no abnormalities. The patient was discharged and referred to his primary care physician for further etiological evaluation of the urinary retention. Subsequently, a renal and bladder ultrasound, including suprapubic and transrectal prostate assessment, revealed a markedly enlarged prostate with irregular contours, with an estimated weight of approximately 385 g, and without differentiation between the central and peripheral zones (Figure 1).

While awaiting a follow-up appointment with his primary care physician, he experienced a new episode of urinary retention, prompting another visit to the ED.

During the second ED visit, the patient presented with suprapubic abdominal pain and discomfort in the context of urinary retention, without accompanying systemic symptoms such as fever, nausea, vomiting, or weight loss. On physical examination, he had suprapubic tenderness on palpation, with a palpable, distended bladder consistent with a bladder globe. The lower abdomen was tense and mildly painful on deep palpation, and he was unable to void.

Based on the clinical history, physical examination, and diagnostic workup, several differential diagnoses were considered, including benign prostatic hyperplasia, prostate neoplasm, prostatitis, retroperitoneal abscess, and pelvic lymphangioma.

**Figure 2:**
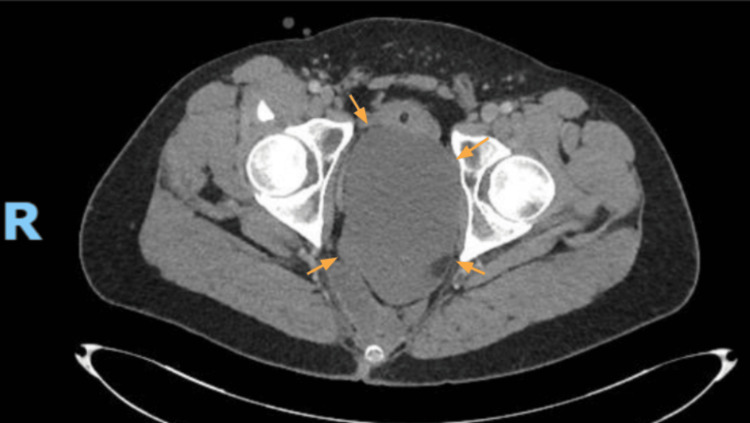
Abdominopelvic axial CT A large, lobulated pelvic mass is identified, measuring 159 × 95 × 88 mm. The lesion demonstrates low attenuation, slightly above that of simple fluid. It exerts a significant mass effect, resulting in anterior and superior displacement of the seminal vesicles and urinary bladder, as well as contouring and rightward displacement of the sigmoid and rectosigmoid colon.

The patient was hospitalized in the Internal Medicine ward for comprehensive evaluation to determine the underlying cause of his condition. During hospitalization, a thoraco-abdomino-pelvic CT scan was performed, revealing a large lobulated pelvic mass with maximum dimensions of 159 × 95 × 88 mm. The lesion had low-density content, slightly higher than that of pure water. The mass exerted a marked mass effect, displacing the seminal vesicles and bladder anterosuperiorly and molding and deviating the sigmoid and rectosigmoid colon to the right. The differential diagnosis included a fluid collection, necrotic mass, lymphangioma, or other lesions (Figure 2).

**Figure 3:**
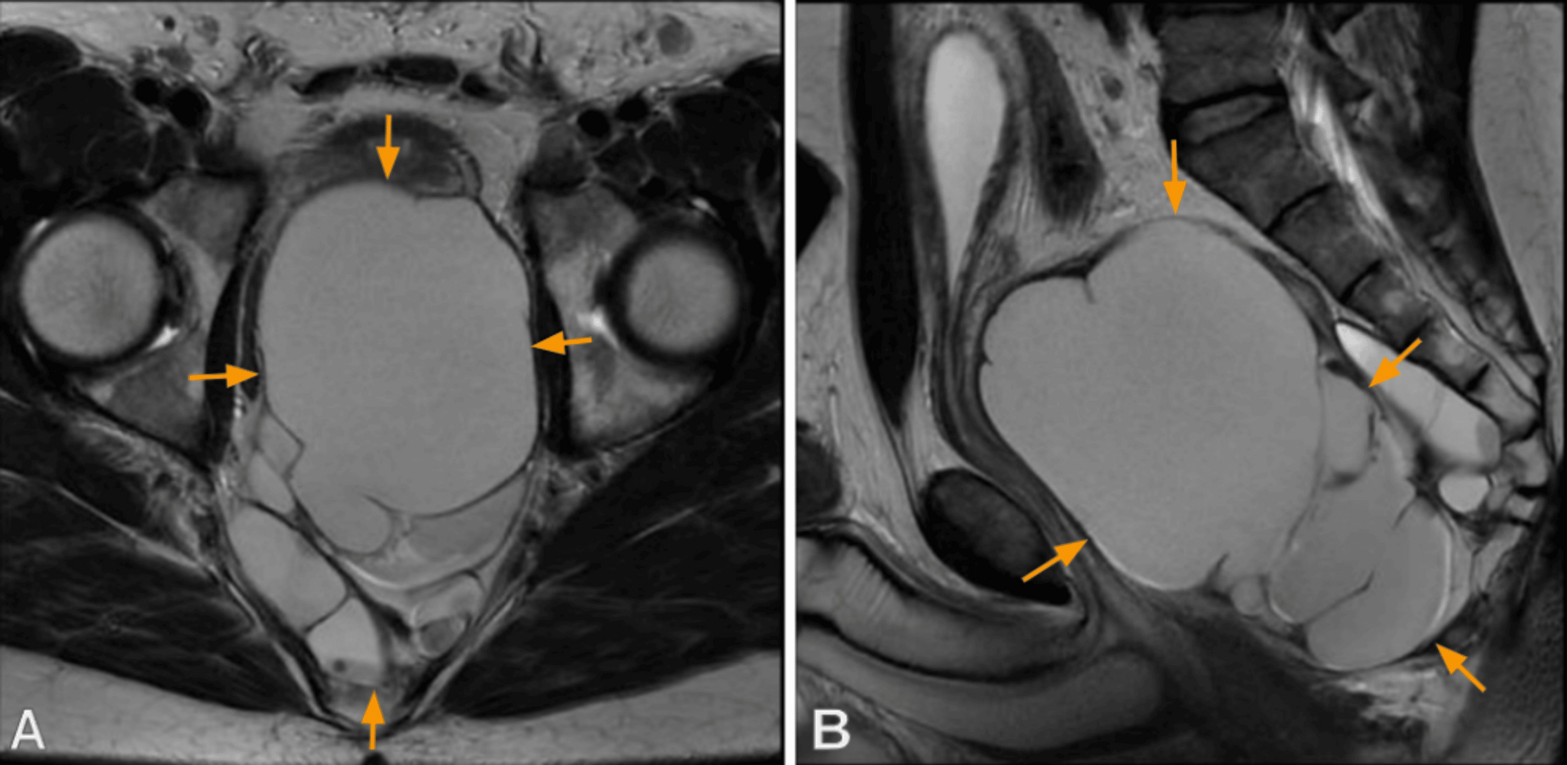
MRI showing (A) axial T2-weighted image and (B) sagittal T2-weighted image The image shows a large, multiloculated cystic mass in the pelvic cavity, with thin, enhancing septa and some parietal calcifications, as well as fluid-filled loci with a high protein content. The lesion pushes against the remaining pelvic organs, without invading them. These findings supported the hypothesis of lymphangioma, measuring 156 × 95 mm (longitudinal (L) × anteroposterior (AP)).

For better characterization, a pelvic MRI was performed, revealing a large, multiloculated cystic mass in the pelvic cavity, with thin enhancing septa, parietal calcifications, and fluid locules with high protein content. The lesion displaced adjacent pelvic organs without evidence of invasion. These findings were compatible with the diagnosis of lymphangioma, measuring 156 × 95 mm (longitudinal (L) × anteroposterior (AP)). The bladder was displaced anteriorly and superiorly by the mass, with no detectable abnormalities. The prostate was displaced anteriorly, also without significant changes on the acquired sequences (Figure 3).

A CT-guided transrectal biopsy of pelvic mass was performed, revealing turbid yellow-brown fluid (Tables 1-2).

**Table 1:** Results of macroscopic and microscopic examination of the biopsy fluid guided by CT

Parameter	Results
Macroscopic examination	
Appearance	Purulent
Color	Whitish
Microscopic examination	
Total cell count (/µL)	Uncountable
Differential count	Predominantly polymorphonuclear cells
Culture	Negative

**Table 2:** Results of chemical examination of the biopsy fluid guided by CT

Parameter	Results	Reference values
pH	7.7	6.8-7.6
Proteins	10.9 g/dL	Transudates < 2; exudates > 2
Albumin	5.7 g/dL	Transudates < 2; exudates > 2
Glucose	<10.0 mg/dL	-
Lactate dehydrogenase (LDH)	1660 U/L	±10% serum activity

**Figure 4:**
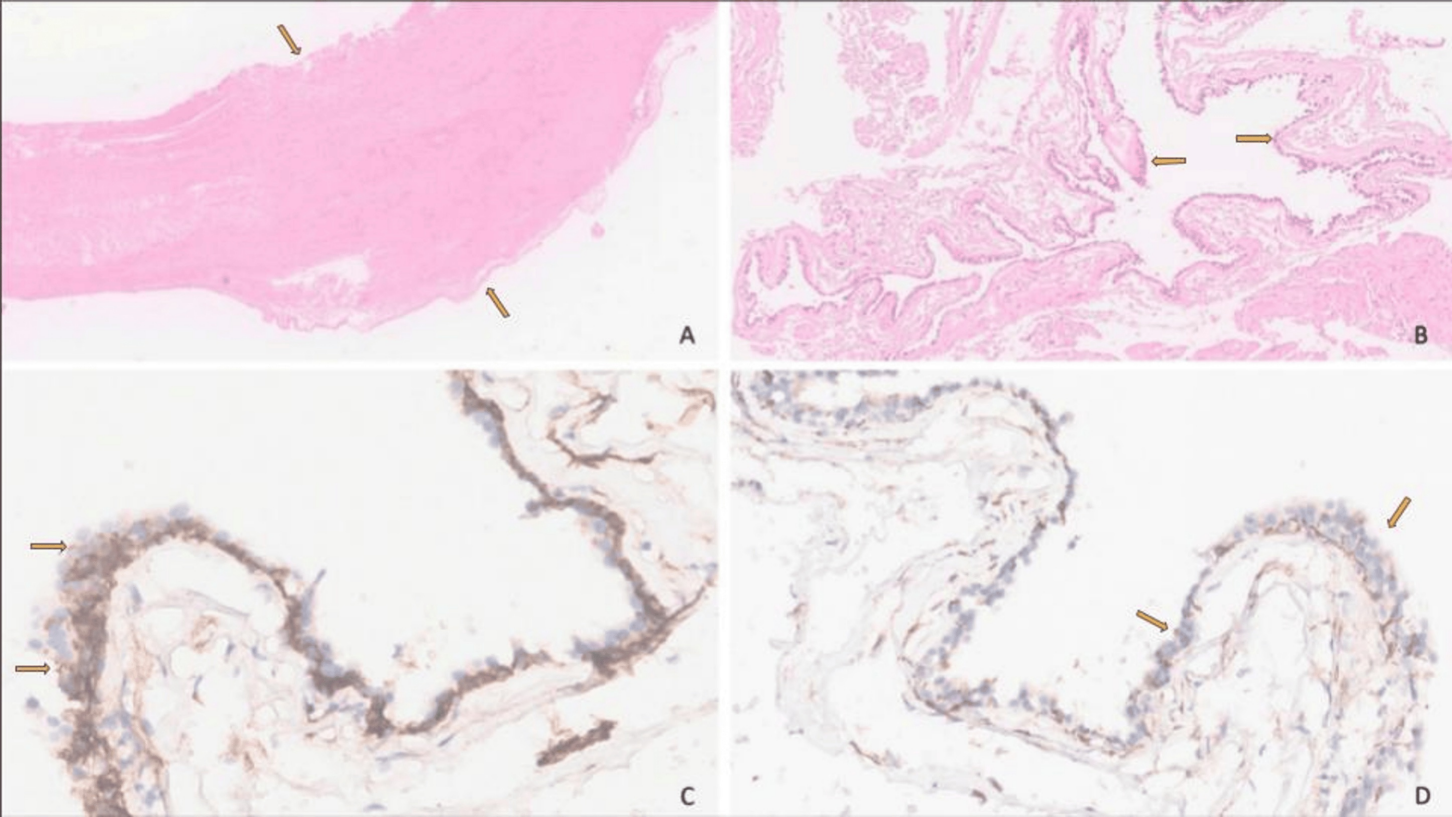
Histopathological examination of the surgical specimen Lymphangioma with a thickened fibrotic cystic wall, (A) partially lined by cells, (B) that express D2-40 (C) and are negative for WT1 (D), compatible with dilated lymphatic channels. (A, B) Hematoxylin and eosin. (C, D) Immunohistochemistry.

The patient underwent elective surgery and showed no complications in the postoperative period. Histopathological examination of the surgical specimen (Figure 4) was characterized by a fibrotic and thickened cystic wall, consistent with a long-standing cystic lymphatic malformation. Portions of the cystic spaces were partially lined by flattened endothelial-type cells. Immunohistochemical staining showed that these lining cells exhibited strong membranous positivity for D2-40, a marker that highlights lymphatic endothelium and confirms their lymphatic nature. In contrast, the cells were negative for WT1, which helps exclude other cystic or vascular proliferations that may express this marker. Taken together, these findings are compatible with dilated lymphatic channels, supporting the diagnosis of lymphangioma.

## Discussion

Pelvic lymphangioma is a rare condition in adults, as most cases are diagnosed in childhood due to its congenital origin. These tumors pose a clinical challenge because symptoms are often nonspecific and result from compression of adjacent structures. Pelvic lymphangiomas account for less than 1% of all lymphangiomas, with an estimated overall incidence of one in 20,000 to 250,000 individuals. Retroperitoneal lymphangiomas, located behind the abdominal cavity, are even rarer, representing fewer than 1% of cases [1-3].

Lymphangiomas are classified in the literature as capillary, cavernous, or cystic, with the cystic type most commonly found in the retroperitoneal and pelvic regions. In our case, the patient presented with a cystic lymphangioma, which was confirmed by histological examination [7-8]. In adults, these lesions may remain asymptomatic for long periods and only become clinically apparent when they reach a size sufficient to cause obstruction, pain, or infectious complications [1,3,7].

Imaging plays a fundamental role in therapeutic planning. In this case, CT and MRI revealed a multiloculated cystic pelvic lesion without signs of invasion of adjacent structures, features typical of lymphangioma. Nevertheless, the differential diagnosis includes cystadenoma, lymphoma, postoperative lymphoceles, retroperitoneal tumors, and mesenchymal neoplasms, making clinical correlation and, in selected cases, histopathological analysis essential [1-3].

The treatment of choice for symptomatic pelvic lymphangioma is complete surgical resection, which reduces the risk of recurrence and complications. However, surgery can be technically challenging due to the proximity of pelvic vessels, ureters, and genitourinary organs [7,9]. When complete resection is not feasible or carries a high risk, image-guided sclerotherapy may be considered as an alternative. In the present case, surgical management allowed adequate disease control, with improvement of urinary symptoms and no evidence of recurrence at follow-up [1,3,7].

## Conclusions

Pelvic lymphangioma, a rare lymphatic vascular malformation in adults, represents a diagnostic challenge due to its nonspecific clinical presentation. In the case described, urinary retention was the initial manifestation, resulting from bladder compression by the cystic mass and highlighting the importance of considering this entity in the differential diagnosis of pelvic masses in adult patients. Imaging, combined with histopathological confirmation, is essential for lesion characterization and the exclusion of other conditions. Complete surgical resection remains the treatment of choice, providing symptom resolution and a low risk of recurrence.

This case contributes to the literature by emphasizing the rarity of pelvic lymphangioma in adults and the need for a multidisciplinary approach to management, underscoring that early identification and appropriate treatment ensure a better prognosis and quality of life for the patient.
